# Circular RNA hsa_circRNA_002178 silencing retards breast cancer progression via microRNA‐328‐3p‐mediated inhibition of COL1A1

**DOI:** 10.1111/jcmm.14875

**Published:** 2020-01-19

**Authors:** Ting Liu, Ping Ye, Yuanyuan Ye, Sen Lu, Baosan Han

**Affiliations:** ^1^ Department of General Surgery Xinhua Hospital Affiliated to Shanghai Jiao Tong University School of Medicine Shanghai China; ^2^ Institute of Biliary Tract Disease Shanghai Jiao Tong University School of Medicine Shanghai China; ^3^ Shanghai Key Laboratory of Biliary Tract Disease Research Shanghai China; ^4^ School of Medical Instrument and Food Engineering University of Shanghai for Science and Technology Shanghai China

**Keywords:** angiogenesis, breast cancer, circular RNA, COL1A1, energy metabolism, Hsa_circRNA_002178, MicroRNA‐328‐3p

## Abstract

Circular RNAs (circRNAs) are a group of non‐coding RNAs implicated in the pathogenesis of cancer progression, which exert their functions via regulation of microRNAs (miRNAs) and genes. The present study uses gain‐ and loss‐of‐function approaches to evaluate the functions of hsa_circRNA_002178 in angiogenesis along with energy metabolism and underlying downstream signals. The expression pattern of hsa_circRNA_002178 in clinical breast cancer tissues and its association with prognosis were characterized at first. Next, the energy metabolism and angiogenesis as well as cell viability were evaluated when the expression of hsa_circRNA_002178 in breast cancer cells was knocked down by siRNA. The interaction between hsa_circRNA_002178 and its downstream miR‐328‐3p was identified, followed by the analysis of their functions in regulation of breast cancer cellular behaviours. The target gene of miR‐328‐3p was predicted and verified, followed by identifying its role in the breast cancer progression. Higher expression of hsa_circRNA_002178 shared an association with worse prognosis in breast cancer. The inhibition of hsa_circRNA_002178 resulted in reductions in cell viability, energy metabolism and tube formation ability. Hsa_circRNA_002178 could competitively bind to miR‐328‐3p and down‐regulated its expression. Restoration of miR‐328‐3p eliminated the tumour‐promoting effects of hsa_circRNA_002178. COL1A1, as a target of miR‐328‐3p, could be up‐regulated by overexpression of hsa_circRNA_002178. In vivo experiments further confirmed the inhibition of tumour growth and inflammation by silencing hsa_circRNA_002178 or up‐regulating miR‐328‐3p. Taken together, hsa_circRNA_002178 is highlighted as a promising target for breast cancer due to the anti‐tumour effects achieved by silencing hsa_circRNA_002178.

## 1 INTRODUCTION

Breast cancer ranks the first commonly diagnosed cancer in female population, with an estimated rate of almost 1 in every 4 female cancer cases in 2018.^1^ Generally, tumour cells exhibit higher capacities of glucose utilization and oxidative metabolism than their non‐tumour equivalents.^2^ It greatly varies in the timing and distribution pattern of tumour metastases. Only a small quantity of patients with metastatic breast cancer has been timely diagnosed.^3^ Successful neoadjuvant chemotherapy with trastuzumab alone or plus pertuzumab portends even greater benefit for the patients with early‐stage HER2‐positive breast cancer.^4^ Even though the treatment options progressed in the recent decades, heterogeneity of metastatic breast cancer and specific molecular mutations in clinical situations have been identified, and personalized strategies are effective for only a few patients up to now.^5^ Strikingly, it is currently acknowledged that circular RNAs (circRNAs) have implications in the pathogenesis of cancer progression, which highlighted their potential as novel biomarkers or therapeutic targets for breast cancer,^6^ this brings an attractive focus to seek circRNAs‐targeted strategies.

CircRNAs are a large group of non‐coding RNAs that exert functions through modulating the activity of microRNAs (miRNAs) and gene expression.^7^ A plenty of circRNAs have been identified by RNA‐seq data in primary breast cancer, and a few of them have been suggested as important predictors, such as circCNOT2 and CREBBP.^8^ Another circRNA hsa_circRNA_001783 has been reported to accelerate breast cancer progression by functioning as a sponge of miR‐200c‐3p.^9^ Additionally, a circRNA circIRAK3 also acts as a promoter of breast cancer metastasis by sponging miR‐3607.^10^ miRNAs are non‐coding RNA molecules that could mediate cell differentiation and survival. Some of them were suggested to be efficacious therapeutic candidates, such as miR‐34 and miR‐122.^11^ Interestingly, miR‐362‐3p and miR‐329 play critical roles as tumour suppressor in the progression of breast cancer via suppression in cellular proliferation and tumour growth.^12^ In this study, hsa_circRNA_002178 is determined to be an up‐regulated circRNA in breast cancer and a circRNA that could bind to miR‐328‐3p. Although it has been suggested that miR‐328‐3p can mediate androgen receptor (AR) in breast cancer cells,^13^ the interaction between hsa_circRNA_002178 and miR‐328‐3p as well as the underlying mechanism is largely unknown. Based on six bioinformatic prediction databases, COL1A1 is identified as a target gene of miR‐328‐3p. Collagen type I alpha 1 chain (COL1A1), encoding type I collagen, associated with oestrogen/progesterone receptor (ER/PR) status, is proposed as a regulator that facilitates breast cancer metastasis.^14^ In this regard, we hypothesized that hsa_circRNA_002178 might regulate the behaviours of breast cancer cells through interacting with in miR‐328‐3p and COL1A1. Both in vitro and in vivo experiments were designed to characterize the role of hsa_circRNA_002178 and further identify the detailed interactions with miR‐328‐3p and COL1A1.

## 2 MATERIALS AND METHODS

### 2.1 Ethics statement

The study was conducted with the approval of the Ethics Committee of Xinhua Hospital Affiliated to Shanghai Jiao Tong University School of Medicine, and written informed consents were obtained from all patients or their guardians. Also, the animal experiments were conducted with the approval of the Animal Ethics Committee of Xinhua Hospital Affiliated to Shanghai Jiao Tong University School of Medicine.

### 2.2 Microarray‐based gene expression analysis

Breast cancer‐related circRNA expression (http://www.ncbi.nlm.nih.gov/geo/query/acc.cgi?acc=GSE101123) and gene expression (http://www.ncbi.nlm.nih.gov/geo/query/acc.cgi?acc=GSE80754 and http://www.ncbi.nlm.nih.gov/geo/query/acc.cgi?acc=GSE10797) datasets were found in Gene Expression Omnibus (GEO) database (https://www.ncbi.nlm.nih.gov/geo/). Differentially expressed circRNAs or genes were analysed using the limma package in R language (http://master.bioconductor.org/packages/release/bioc/html/limma.html). A differential gene expression heat map was plotted using pheatmap package (https://cran.r-project.org/web/packages/pheatmap/index.html). The threshold value for screening differentially expressed genes was set as *P* < .05 and |log_2_ (fold change)| > 1. Downstream miRNAs of differentially expressed circRNA candidates were predicted in two databases circBank (http://www.circbank.cn/index.html) and starBase (http://starbase.sysu.edu.cn/index.php) and intersected using jvenn software (http://jvenn.toulouse.inra.fr/app/example.html). Next, starBase, RNA22 (https://cm.jefferson.edu/rna22/), mirDIP (http://ophid.utoronto.ca/mirDIP/), miRWalk (http://mirwalk.umm.uni-heidelberg.De/), miRmap (https://mirmap.ezlab.org/) and TargetScan (http://www.targetscan.org/vert_71/) were applied to predict target genes of miRNA candidates. The putative target genes are intersected with the differentially expressed genes and selected as candidate. In addition, the expression of miRNAs/genes in breast cancer in the Pan‐Cancer Analysis Platform of starBase was analysed.

### 2.3 Clinical sample collection

Breast cancer and adjacent normal tissues (at least 5 cm from the edge of breast cancer tissues) were obtained from 70 patients who were diagnosed with breast cancer between 2016 and 2018. The inclusion criteria included (1) female patients; (2) patients pathologically diagnosed with breast cancer; (3) patients who had not received any chemotherapy, radiotherapy or immunotherapy prior to the operation; and (4) patients who had complete clinical and pathological data. The exclusion criteria included (1) male patients; (2) female patients at gestation period or lactation period; and (3) patients with other tumours. The follow‐up visit lasted for 6‐36 months. There were 49 patients aged ≥40 years and 21 years patients aged <40 years. The diameters of tumours were >2 cm in 44 cases, and ≤2 cm in 26 cases. Tissue samples were freshly frozen in liquid nitrogen and stored in a −80°C refrigerator for later use.

### 2.4 Cell line selection

The immortalized human mammary epithelial cell line MCF‐10A and breast cancer cell lines (BT549, MCF‐7, MDA‐MB‐231 and T47D) and human umbilical vein endothelial cells (HUVECs) used in this study were purchased from American Type Culture Collection (ATCC) (https://www.atcc.org/). MCF‐10A was incubated with dulbecco's modified eagle medium (DMEM)/F12 (1:1) containing 5% horse serum, 20 ng/mL epidermal growth factor (EGF), 10 μg/mL insulin + 0.5 μg/mL hydrocortisone and 100 U/mL penicillin/streptomycin at 37°C with 5% CO_2_. MCF‐7 and MDA‐MB‐231 were incubated with 90% DMEM‐H + 10% foetal bovine serum (FBS) (10569044, Gibco BRL/Invitrogen) at 37°C with 5% CO_2_. BT549 and T47D were seeded in Roswell Park Memorial Institute (RPMI)‐1640 medium (11875093; Gibco BRL/Invitrogen) containing 10% FBS (10100147; Gibco BRL/Invitrogen) supplemented with 100 U/mL penicillin/streptomycin (15140122; Gibco BRL/Invitrogen) and cultured at 37°C with 5% CO_2_. The expression of hsa_circRNA_002178 in each cell line was determined by reverse transcription quantitative polymerase chain reaction (RT‐qPCR), and the cell line with the highest expression was selected for subsequent experiments.

### 2.5 Cell treatment

miR‐328‐3p mimic, lentivirus vector of miR‐328‐3p overexpression (oe‐miR‐328‐3p), hsa_circRNA_002178 or COL1A1 wild‐type sequence containing miR‐328‐3p binding site (hsa_circRNA_002178‐wt or COL1A1‐wt), hsa_circRNA_002178 or COL1A1 mutant type sequence with mutated miR‐328‐3p binding site (hsa_circRNA_002178‐mut or COL1A1‐mut) and negative control of mimic (mimic NC) were constructed based on the G418‐resistant pSilencer 4.1‐CMV neo and Pegfp‐4.1N vector purchased from the Sangon Biotechnology Co. Ltd. siRNA targeting hsa_circRNA_002178 (si‐hsa_circRNA_002178; si‐hsa_circRNA_002178‐1: CTGAGCTTCGGGGAGCTGAGT; si‐hsa_circRNA_002178‐2: GCTTCGGGGAGCTGAGTGCGT) and negative control siRNA (si‐NC) were from Mission RNAi (Sigma‐Aldrich Chemical Company). Meanwhile, the lentiviral blank control of hsa_circRNA_002178 overexpression (oe‐NC) and the lentiviral vector overexpressing hsa_circRNA_002178 (oe‐hsa_circRNA_002178) were constructed. The full‐length hsa_circRNA_002178 cDNA was cloned into pCDH‐CMV‐MCS‐EF1‐GFP + Puro to obtain the overexpression vector of hsa_circRNA_002178.

Lipofectamine™ 2000 reagent (11668019; Invitrogen) was utilized for transfection of 50 nmol/L miR‐328‐3p mimic or mimic NC into cells, and Lipofectamine™ RNA iMAX reagent (13778030; Invitrogen) was for transfection of siRNA into cells.

### 2.6 Fluorescence in situ hybridization (FISH)

The subcellular localization of hsa_circRNA_002178 was identified by FISH kit (BIS‐P0001, Guangzhou Bersin Biotechnology Co., Ltd.). The breast cancer cells cultured after transfection were seeded into slides. The slides were baked at 50°C for 2‐3 hours, denatured in 2 × sodium citrate buffer (SSC) for 2‐3 minutes and followed by gradient dehydration with a volume fraction of 70%, 85% and 95% ethanol (3 minutes each time). The air‐dried slides were hybridized with hsa_circRNA_002178 probe (Table S1) hybridization solution labelled with digoxigenin at 42°C for 16 hours, and the hsa_circRNA_002178 antagonist probe (Table S1) (Invitrogen) was used as a NC. After hybridization, the slides were immersed in 2 × SSC 3 times (5 minutes each time) and soaked in 70% ethanol for 3 minutes. After that, the naturally dried slides were stained with 4′6‐diamidino‐2‐phenylindole (DAPI) for 5‐10 minutes in the dark and then washed twice with ice‐cold PBS. The fluorescence images were acquired under a laser confocal scanning microscope coupled with Zeiss LSM880 NLO confocal microscope system (Leica Microsystems).

### 2.7 RNA extraction and quantification

Total RNA was extracted from tissues or cells using the TRIzol kit (10296010, Invitrogen) 36 hours after transfection. The primers used were all synthesized by Beijing Genomics Institute (BGI) group (Table 1). miRNA‐specific complementary DNA was synthesized using TaqMan™ MicroRNA Reverse Transcription Kit (4366596; Applied Biosystems). The expression of miR‐328‐3p was determined by TaqManTM MicroRNA Assay Quantitative PCR Kit (4427975, Applied Biosystems). The cDNA templates of genes were synthesized using a PCR instrument according to the instructions of EasyScript First‐Strand cDNA Synthesis SuperMix kit (AE301‐02; TransGen Biotech Inc). The quantitative PCR was conducted using SYBR® Premix Ex Taq™ II kit (RR820A, TaKaRa) in ABI 7500 real‐time PCR instrument (Applied Biosystems). The relative expression of genes or miR‐328‐3p was calculated according to the 2^−∆∆Ct^ method. The expression of genes was normalized to GAPDH and that of miR‐328‐3p was normalized to U6.

**Table 1:** Sequences for reverse transcription quantitative polymerase chain reaction

Gene	Sequence
miR‐328‐3p	F: 5′‐TGCGGCTGGCCCTCTCTGCCC‐3′
	R: 5′‐CCAGTGCAGGGTCCGAGGT‐3′
U6	F: 5′‐AAAGCAAATCATCGGACGACC‐3′
	R: 5′‐GTACAACACATTGTTTCCTCGGA‐3′
hsa_circRNA_002178	F: 5′‐CACTCCACTCCCATGTCCC‐3′
	R: 5′‐GCCTCTGGCCCTAGTCTCA‐3′
COL1A1	F: 5′‐TCTAGACATGTTCAGCTTTGTGGAC‐3′
	R: 5′‐TCTGTACGCAGGTGATTGGTG‐3′
GAPDH	F: 5′‐GGCGTTCTCTTTGGAAAGGTGTTC‐3′
	R: 5′‐GTACTCAGCGGCCAGCATCG‐3′
si‐NC	GGATCCCACCTTAAGGACTG
si‐hsa_circRNA_002178‐1	CTGAGCTTCGGGGAGCTGAGT
si‐hsa_circRNA_002178‐2	GCTTCGGGGAGCTGAGTGCGT
mimic NC	UUCUCCGAACGUGUCACGUTT
miR‐328‐3p‐mimic	CUGGCCCUCUCUGCCCUUCCGU

Abbreviations: COL1A1, collagen type I alpha 1 chain; F, forward; GAPDH, glyceraldehyde‐3‐phosphate dehydrogenase; miR‐328‐3p, microRNA‐328‐3p; R, reverse.

### 2.8 Cell counting kit (CCK‐8)

Breast cancer cell proliferation was performed by CCK‐8 method (CK04, Dojindo Laboratories). The cells were seeded in a 96‐well plate at a cell density of 2000 cells per well and cultured with 100 μL of RPMI‐1640 at 37°C with 5% CO_2_. The experiment was conducted in at least three duplicate wells. The cells were added with 10 μL of CCK‐8 reagent at 37°C with 5% CO_2_ at the 0th, 24th, 48th, 72nd and 96th hours and incubated for another 4 hours. Optical density (OD) value of each well at a wavelength of 450 nm was measured on a microplate reader.

### 2.9 Western blot analysis

Cells were lysed on ice by 1 mL immunoprecipitation (IP) lysis buffer (P0013; Beyotime Biotechnology Co. Ltd.) containing 10 μL PMSF (100 mmol/L) (ST506; Beyotime Biotechnology Co. Ltd.). The proteins were separated by sodium dodecyl Sulfatepolyacrylamide gel electrophoresis (SDS‐PAGE). The separated proteins were transferred to a polyvinylidene fluoride (PVDF) membrane (FFP36; Beyotime Biotechnology Co. Ltd.) and blocked with 5% bovine serum albumin (BSA) for 2 hours at 37°C. The membranes were incubated with 0.125 μg/mL of mouse anti‐GAPDH monoclonal antibody (A21994; Invitrogen), 1 μg/mL of mouse anti‐human PCNA monoclonal antibody (ab29; Abcam), rabbit anti‐human Ki67 monoclonal antibody (ab92742, 1:1000; Abcam) and rabbit anti‐human COL1A1 polyclonal antibody (GTX112731, 1:1000; Genetex) overnight at 4°C. The membrane was washed with 0.1% TBST 3 times for 10 minutes. Goat antimouse IgG (ab205719, 1:20 000, Abcam) or goat anti‐rabbit IgG (ab6721, 1:20 000, Abcam) was applied as secondary antibody. The membrane was developed by developing agent (P0020; Beyotime). Protein bands were imaged by a Bio‐Rad gel imaging system, and protein expression relative to GAPDH was determined by Image J software (NIH).

### 2.10 Basal oxygen consumption rate (OCR) and extracellular acidification rate (ECAR) measurement

The OCR and ECAR were determined by Seahorse XF24 analyzer (Agilent Technologies) as previously described.^15^ The cells were cultured in high‐glucose DMEM containing 10% FBS and 5% glutamine.

For determination of OCR, 100 μL of cell suspension was seeded into 24‐well Seahorse XF24 plate (100882‐004, Agilent Technologies) 24 hours before experiment with 4 × 10^5^ per well. The experiments were conducted in 4 replicates per group. In addition, four wells with medium only were used for background subtraction. In order to minimize the edge effect of plating and to ensure the formation of monolayer cells at the bottom of the wells, the microplates were covered with a cell culture hood for 1 hours at room temperature and then placed in a 37°C, 5% CO_2_ incubator. After the cells were attached to the plate (about 3 hours), cells were cultured with medium (150 μL/well) overnight. The medium was replaced with 675 μL Seahorse medium 1 hour before the experiment. The cells were then incubated for 1 hour in a 37°C incubator without CO_2_. The XF24 sensor cartridge was hydrated overnight in a XF calibration solution in a 37°C incubator without CO_2_.

For determination of ECAR, 75 μL of 15 μmol/L ATP synthase inhibitor (oligomycin), 83 μL of 5 μmol/L carbonyl cyanide trifluoromethoxyphenylhydrazine (FCCP, mitochondrial inner membrane uncoupler) and 92 μL of 10 μmol/L antimycin A (complex III inhibitor) and 2 μmol/L rotenone (complex I inhibitor) were loaded onto three respective ports of the sensor cartridge before the experiment. For background subtraction, Seahorse Run Media was loaded into each port instead of the original solution. The sensor cartridge was loaded into the Seahorse XF24 analyzer and calibrated. After calibration of the sensor box, the experiment was conducted with microplate loaded onto the machine.

### 2.11 High‐performance liquid chromatography (HPLC)

Cellular energy metabolism analysis was performed using SHIMADZU Shimadzu ultra‐fast liquid chromatography to detect the contents of ATP, ADP, AMP, NADH and NAD. The treated cell samples were reacted in an Agela Venusil MP C18 column (250 mm × 4.6 mm, 5 μm) (VA952505‐0, Tianjin Bona Aigel Technology Co., Ltd.). The mobile phase consisted of two phases, A and B. The mobile phase A consisted of 50 mmol/L disodium hydrogen phosphate and 15 mmol/L trimethylamine (TEA). The pH was adjusted to 7 with acetic acid (HAc). The mobile phase B was methanol. Reversed phase ion‐pair HPLC was used to isolate the target compound in the cells using an isocratic elution method [4% (v/v) methanol]. The flow rate used here was 0.8 mL/min, and 20 μL volume samples or standards were loaded at room temperature and analysed by dual‐wavelength spectrophotometry at 254 nm and 266 nm.

### 2.12 Tube formation assay

Human umbilical vein endothelial cells and MDA‐MB‐231 cells were separately cultured with DMEM containing 10% FBS and RPMI‐1640 in a 37°C incubator. After 48 hours of transfection, the cells were centrifuged and tumour cell supernatant was collected. The tumour cell supernatant, DMEM and FBS were mixed at a ratio of 4:5:1. Each well was added with 50 μL Matrigel in a 96‐well culture plate and gelled in a 37°C incubator for 30 minutes. After coagulation, tumour cell–conditioned medium and HUVEC suspension (2.5 × 10^5^ cells/well) were mixed and co‐cultured for 8 hours under conditions of 37°C and 5% CO_2_. Four fields of view were selected under the phase contrast microscope (CK40, Olympus). The total length and number of nodes were quantified and photographed.

### 2.13 RNA pull‐down

The binding of hsa_circRNA_002178 to miR‐328‐3p was analysed using the Magnetic RNA‐Protein Pull‐Down kit (20164; Pierce/Thermo Fisher Scientific). After detachment of breast cancer cells, the cells were lysed by RNA immunoprecipitation (RIP) lysis buffer on ice for 2 minutes and then centrifuged at 4°C for 10 minutes. The cell lysate was incubated with Bio‐miR‐328‐3p (GACCGGGAGAGACGGGAAGGCA) and biotinylated Bio‐NC (GCACTTTAGCGCCAG ATTATCG) (Pierce™ RNA 3′ End Desthiobiotinylation Kit, 20163, Thermo Fisher Scientific) and streptavidin‐labelled magnetic beads overnight at 4°C. Finally, RNA was extracted by the TRIzol method and then subjected to RT‐qPCR to determine the enrichment of hsa_circRNA_002178.

### 2.14 Dual luciferase reporter assay

The miR‐328‐3p putative binding sites were predicted using RNA22 biological prediction website (https://cm.jefferson.edu/rna22/Interactive/). Based on the predicted binding sites, the synthesized full‐length hsa_circRNA_002178 and COL1A1 mRNA 3′‐UTR fragments were inserted into the pGL3‐basic vector (P2129; Shanghai HeWu Biotechnology Co., Ltd.). The vector structure was shown in the Appendix S1 and Appendix S2. Wild‐type (wt) reporter plasmids named hsa_circRNA_002178‐wt and COL1A1‐wt were obtained. A complementary sequence contained mutated binding site was constructed and inserted into the reporter vector to construct hsa_circRNA_002178‐mut and COL1A1‐mut plasmids. The correctly sequenced luciferase reporter plasmids were cotransfected into breast cancer cells with miR‐328‐3p mimic or mimic NC, respectively. The luciferase activity was determined using a dual luciferase assay kit (E1910; Promega) on a Promega GLoma × 20/20 Luminometer (E5311; purchased from Zhongmei Biotechnology Co., Ltd.). The experiments were repeated three times.

### 2.15 Tumour xenografts in nude mice

Breast cancer cells were stably transfected with si‐hsa_circRNA_002178, si‐NC, oe‐hsa_circRNA_002178 and/or oe‐miR‐328‐3p (lentivirus vector of miR‐328‐3p overexpression) or oe‐NC (NC of miR‐328‐3p lentivirus). About 1 × 10^7^ cells were subcutaneously injected into axilla of female athymic specific‐pathogen‐free (SPF) BALB/C nude mice (aged 4‐5 weeks; weighing 16‐20 g; purchased from the Shanghai Laboratory Animal Center of the Chinese Academy of Sciences (Shanghai, China). Tumour growth was monitored weekly by measuring the width (W) and length (L) with a caliper, and the volume (V) of the tumour was calculated using the formula: *V* = (*W* ^2^ × *L*)/2. Four weeks after the injection, the mice were killed and the tumour tissues were excised. The tumours were weighed and photographed. The tumours were fixed with a 4% paraformaldehyde and then frozen in liquid nitrogen, which were stored at −80°C for subsequent study.

### 2.16 Immunohistochemistry

The excised mouse tumour tissues were fixed in 4% paraformaldehyde and embedded in paraffin, and sliced into 5‐μm‐thick sections. Paraffin‐embedded sections were deparaffinized and rehydrated, and then subjected to antigen retrieval in 0.01 mol/L citrate buffer at 95°C for 30 minutes. The sections were then incubated overnight at 4°C with antibodies to IL‐6 (BS6419; 1:100, Bioworld) and TNF‐α (GTX110520, 1:100, Gene Tex) or COL1A1 (GTX112731, 1:500, Gene Tex). The sections were then treated with diluted goat anti‐rabbit IgG (ab6795, Abcam). With avidin‐conjugated horseradish peroxidase (HRP) and diaminobenzidine (DAB) as substrate, the sections were counter‐stained with haematoxylin. The images were analysed using Nikon image analysis software (Nikon).

### 2.17 Enzyme‐linked immunosorbent assay (ELISA)

Blood was collected from the ocular venous plexus of mice after deep anaesthesia and allowed to stand at 4°C for 1 hour. The serum was collected after centrifugation at 700 *g* and then stored at −80°C for subsequent use. The levels of inflammatory factors IL‐6 and TNF‐α were measured by Simple Step ELISA^®^ Mouse Kits for IL‐6 (ab100712) and TNF‐α (ab208348) form Abcam, and the OD value was read at 450 nm using an EON spectrophotometer (BioTek Instruments).

### 2.18 Statistical analysis

All data were processed using SPSS 21.0 statistical software (IBM). All data were tested for normal distribution and homogeneity. Measurement data were expressed by mean ± standard deviation. Data were compared using unpaired *t* test. Comparison between groups was performed by one‐way analysis of variance (ANOVA) with Tukey post hoc test. Data at multiple time points were compared by repeated measurement ANOVA. A value of *P* < .05 indicates that the difference is statistically significant.

## 3 RESULTS

### 3.1 The differential expression pattern of hsa_circRNA_002178, miR‐328‐3p and COL1A1 in breast cancer

**Figure 1:**
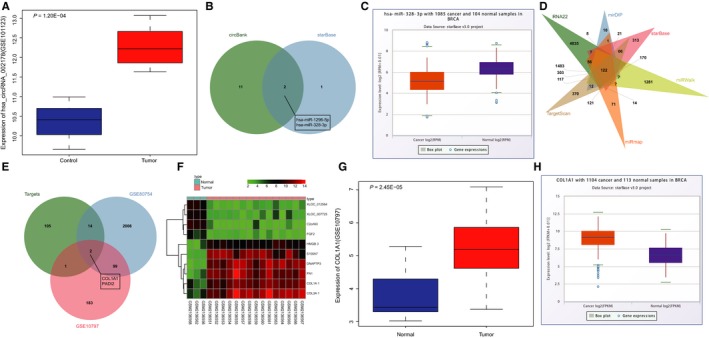
The expression pattern of hsa_circRNA_002178, miR‐328‐3p and COL1A1 in breast cancer. A, the dysregulation of hsa_circRNA_002178 in breast cancer shown by microarray data http://www.ncbi.nlm.nih.gov/geo/query/acc.cgi?acc=GSE101123. B, intersection miRNAs that could be regulated by hsa_circRNA_002178 predicted by circBank and starBase databases. C, the expression of miR‐328‐3p in breast cancer obtained from the Pan‐Cancer Analysis Platform of starBase database. D, intersection target genes of miR‐328‐3p predicted from RNA22, mirDIP, starBase, miRWalk, miRmap and TargetScan databases. E, Target genes of miR‐328‐3p intersected with differentially expressed genes in microarray data (http://www.ncbi.nlm.nih.gov/geo/query/acc.cgi?acc=GSE80754 and http://www.ncbi.nlm.nih.gov/geo/query/acc.cgi?acc=GSE10797). F, heatmap of 10 differentially expressed genes with largest fold change in breast cancer in microarray data (http://www.ncbi.nlm.nih.gov/geo/query/acc.cgi?acc=GSE80754). G, COL1A1 expression in http://www.ncbi.nlm.nih.gov/geo/query/acc.cgi?acc=GSE10797. H, COL1A1 expression in the Pan‐Cancer Analysis Platform

Differentially expressed circRNAs were screened from circRNA expression microarray data of breast cancer (http://www.ncbi.nlm.nih.gov/geo/query/acc.cgi?acc=GSE101123), which showed that hsa_circRNA_002178 expression in breast cancer tissue was notably higher than in the non‐tumour breast tissues (Figure 1A). hsa_circRNA_002178 is also called hsa_circRNA_0000519 in the circBase database (http://www.circbase.org/). The downstream miRNAs of hsa_circRNA_002178 were then predicted in databases circBank and starBase, and two intersection miRNAs were found in the predicted miRNAs from the two databases, named hsa‐miR‐1296‐5p and hsa‐miR‐328‐3p (Figure 1B). It was further suggested that miR‐328‐3p was underexpressed in breast cancer in the Pan‐Cancer Analysis Platform of starBase database (Figure 1C), suggesting that hsa_circRNA_002178 might function in the progression of breast cancer through regulating miR‐328‐3p. To further analyse the possible regulatory mechanisms of miR‐328‐3p, the target genes of miR‐328‐3p were predicted in RNA22, mirDIP, starBase, miRWalk, miRmap and TargetScan databases. The predicted target genes were compared, and Venn maps of those genes were plotted. As shown in Figure 1D, there were 122 intersection target genes that were highly likely to be regulated by miR‐328‐3p. In addition, differentially expressed genes were obtained from gene expression microarray data (http://www.ncbi.nlm.nih.gov/geo/query/acc.cgi?acc=GSE80754 and http://www.ncbi.nlm.nih.gov/geo/query/acc.cgi?acc=GSE10797), and these differentially expressed genes were intersected with the predicted target genes of miR‐328‐3p. Two intersection genes COL1A1 and PADI2 were identified (Figure 1E). COL1A1 was observed to be highly expressed in breast cancer in both http://www.ncbi.nlm.nih.gov/geo/query/acc.cgi?acc=GSE80754 (Figure 1F) and http://www.ncbi.nlm.nih.gov/geo/query/acc.cgi?acc=GSE10797 (Figure 1G), whereas PADI2 was lowly expressed. In addition, the high expression of COL1A1 in breast cancer was verified in the Pan‐Cancer Analysis Platform (Figure 1H). Therefore, we hypothesized that the dysregulation of COL1A1 in breast cancer may be regulated by miR‐328‐3p.

### 3.2 High expression of hsa_circRNA_002178 in breast cancer is associated with poor prognosis

**Figure 2:**
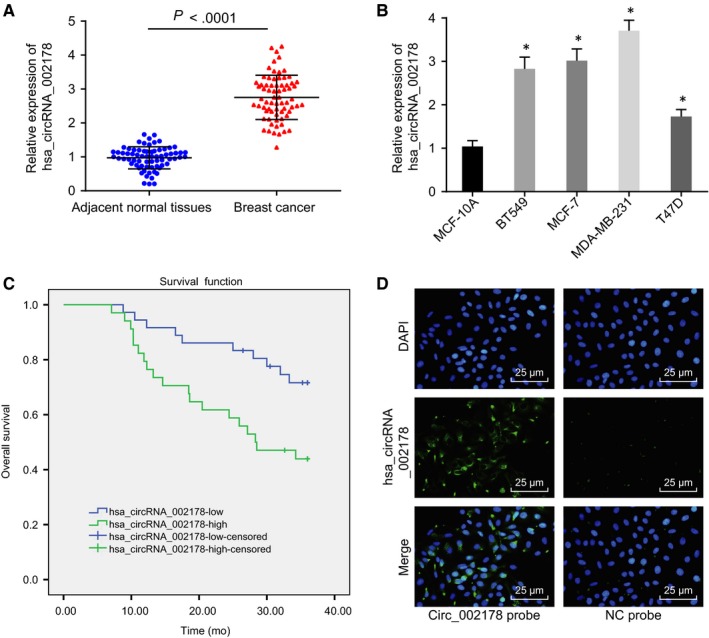
Hsa_circRNA_002178 was highly expressed in breast cancer tissues and cells, which was associated with poor prognosis. A, the expression of hsa_circRNA_002178 normalized to GAPDH in breast cancer and adjacent normal tissues determined by RT‐qPCR, n = 70. B, the expression of hsa_circRNA_002178 normalized to GAPDH in breast cancer cell lines and mammary epithelial cell line determined by RT‐qPCR. C, survival rates of breast cancer patients with high or low hsa_circRNA_002178 expression by Kaplan‐Meier analysis. D, localization of hsa_circRNA_002178 in breast cancer cells (×400). ^*^ *P* < .05 vs adjacent normal tissues or normal cells. The measurement data were expressed as mean ± standard deviation. Data between the two groups were analysed by unpaired *t* test, and among multiple groups were analysed using one‐way ANOVA. The cell experiments were repeated three times

To investigate the involvement of hsa_circRNA_002178 in breast cancer, we first determined hsa_circRNA_002178 expression in 70 breast cancer tissues and their matched adjacent normal tissues, as well as that in breast cancer cell lines (MDA‐MB‐231, BT‐20, MCF‐7 and T47D) and the human mammary epithelial cell line MCF‐10A by RT‐qPCR. The results showed that the expression of hsa_circRNA_002178 was higher in breast cancer tissues than in the adjacent normal tissues (*P* < .05) (Figure 2A). Meanwhile, compared with MCF‐10A, hsa_circRNA_002178 was expressed at a higher level in 4 breast cancer cell lines (*P* < .05) (Figure 2B), wherein the difference was highest in MDA‐MB‐231 cells, so subsequent experiments were performed using MDA‐MB‐231 cells. Next, correlation between high hsa_circRNA_002178 expression with cancer prognosis was investigated with the use of Kaplan‐Meier analysis, which displayed that the survival rate of breast cancer patients with high expression of hsa_circRNA_002178 was remarkably lower than that of patients with low expression of hsa_circRNA_002178 (*P* < .05) (Figure 2C). The above experiments demonstrated that hsa_circRNA_002178 was highly expressed in breast cancer tissues and cells and associated with prognosis in breast cancer patients. Additionally, FISH was utilized to identify its subcellular localization of hsa_circRNA_002178, which showed that hsa_circRNA_002178 was localized in the cytoplasm of breast cancer cells (Figure 2D).

### 3.3 Silencing of hsa_circRNA_002178 impairs breast cancer cell proliferation, energy metabolism and angiogenesis

**Figure 3:**
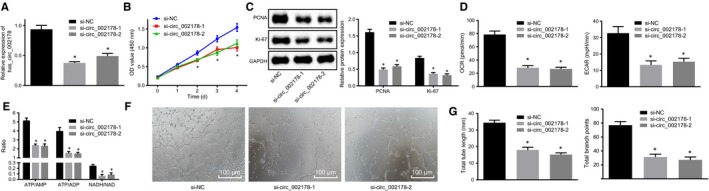
Silencing hsa_circRNA_002178 contributed to suppression in the proliferation, energy metabolism and angiogenesis of breast cancer cells. MDA‐MB‐231 cells were transfected with either si‐hsa_circRNA_002178‐1 or si‐hsa_circRNA_002178‐2 to silence its expression, with si‐NC used as control. A, the expression of hsa_circRNA_002178 determined by RT‐qPCR. B, the viability of breast cancer cells assessed by CCK‐8. C, PCNA and Ki67 protein expression normalized to GAPDH measured by Western blot assay. D, ECAR and OCR levels in MDA‐MB‐231 cells measured by Seahorse XF24 analyzer. E, energy metabolism of breast cancer cells measured by HPLC. F, angiogenesis of MDA‐MB‐231 cells (×100). G, the tube length and the number of nodes. ^*^ *P* < .05 vs the si‐NC group (MDA‐MB‐231 cells transfected with si‐NC). The measurement data were expressed as mean ± standard deviation. Data among multiple groups were analysed using one‐way ANOVA. The cell experiment was repeated three times

Given expression pattern of hsa_circRNA_002178 in breast cancer, the potential function of hsa_circRNA_002178 was examined by silencing hsa_circRNA_002178 in the MDA‐MB‐231 cells. RT‐qPCR data exhibited that the expression of hsa_circRNA_002178 was successfully knocked down after transfection with either si‐hsa_circRNA_002178‐1 or si‐hsa_circRNA_002178‐2 (*P* < .05) (Figure 3A). Next, the results of CCK‐8 and Western blot assay showed that the viability of breast cancer cells transfected with either si‐hsa_circRNA_002178‐1 or si‐hsa_circRNA_002178‐2 was inhibited, accompanied with reductions in expression of proliferation‐related proteins PCNA and Ki67 (*P* < .05) (Figure 3B,C). At the same time, the results obtained from Seahorse XF24 analyzer and HPLC exhibited that the levels of energy metabolism markers ECAR, OCR, ATP and their metabolites of breast cancer cells were reduced by transfection with either si‐hsa_circRNA_002178‐1 or si‐hsa_circRNA_002178‐2 (*P* < .05) (Figure 3D,E). The results of tubular formation experiments showed that the angiogenic ability of breast cancer cells after transfection with either si‐hsa_circRNA_002178‐1 or si‐hsa_circRNA_002178‐2 was attenuated (*P* < .05) (Figure 3F,G). Considering si‐hsa_circRNA_002178‐1 had the highest knockdown efficiency, it was used in the following experiments. The above experimental data suggested that inhibition of hsa_circRNA_002178 expression impeded the proliferation, energy metabolism and angiogenesis of breast cancer cells.

### 3.4 Hsa_circRNA_002178 binds to miR‐328‐3p

**Figure 4:**
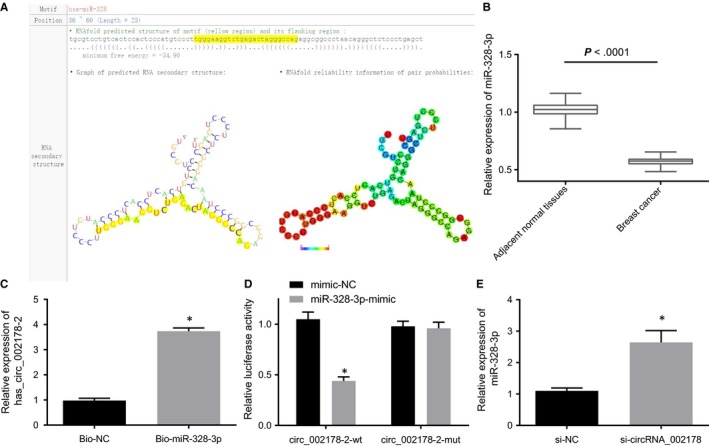
The interaction between hsa_circRNA_002178 and miR‐328‐3p. A, bioinformatics analysis of binding sequence between hsa_circRNA_002178 and miR‐328‐3p. B, determination of miR‐328‐3p expression in breast cancer tissues and their matched adjacent normal tissues by RT‐qPCR, n = 70. C, RNA pull‐down assay for identifying the interaction between hsa_circRNA_002178 and miR‐328‐3p. D, the luciferase activity of hsa_circRNA_002178‐wt or hsa_circRNA_002178‐mut in the presence of miR‐328‐3p; E, determination of miR‐328‐3p expression in breast cancer cells after silencing of hsa_circRNA_002178 by RT‐qPCR. ^*^ *P* < .05 vs the Biotin NC, mimic NC or si‐NC group (cells transfected with Biotin NC, mimic NC or si‐NC). The measurement data were expressed as mean ± standard deviation. Data between the two groups were analysed by unpaired *t* test, and among multiple groups were analysed using one‐way ANOVA. The experiment was repeated three times

Prediction results from circBank and starBase databases found that hsa_circRNA_002178 may bind to miR‐328‐3p in breast cancer cells. As shown in Figure 4A, complementary sequences between hsa_circRNA_002178 and miR‐328‐3p were predicted to be potential binding sites. The expression of miR‐328‐3p in breast cancer tissues was lower than that in the matched adjacent normal tissues determined by RT‐qPCR (*P* < .05; Figure 4B). The results of the RNA pull‐down experiment showed that the biotinylated miR‐328‐3p‐enriched hsa_circRNA_002178 content was significantly increased compared with the biotinylated NC‐enriched one (*P* < .05; Figure 4C). The miR‐328‐3p mimic was transfected into the breast cancer cells expressing hsa_circRNA_002178‐wt or hsa_circRNA_002178‐mut for dual luciferase reporter assay. The results showed that only the luciferase activity of the hsa_circRNA_002178‐wt was decreased by miR‐328‐3p mimic transfection, indicating that miR‐328‐3p specifically bound to hsa_circRNA_002178 (*P* < .05) (Figure 4D). Meanwhile, RT‐qPCR results displayed that miR‐328‐3p expression was increased in breast cancer cells after silencing hsa_circRNA_002178 (*P* < .05; Figure 4E). The above experiments demonstrated that hsa_circRNA_002178 was capable of binding to miR‐328‐3p.

### 3.5 Hsa_circRNA_002178 facilitates cancer progression in vitro through inhibiting miR‐328‐3p

**Figure 5:**
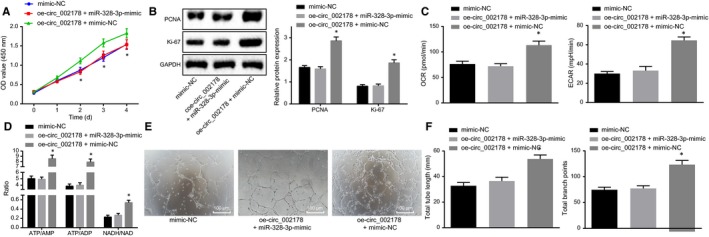
Hsa_circRNA_002178 decreases miR‐328‐3p expression to regulate energy metabolism and angiogenesis. miR‐328‐3p mimic and hsa_circRNA_002178 overexpression plasmid or their NCs were utilized to alter the expression of miR‐328‐3p and/or hsa_circRNA_002178 in MDA‐MB‐231 cells. A, the proliferative capacity of MDA‐MB‐231 cells assessed by CCK‐8 method. B, protein bands imaged using Image J software and the expression of PCNA and Ki67 in MDA‐MB‐231 cells quantified by Western blot analysis; C, ECAR and OCR levels in MDA‐MB‐231 cells measured by Seahorse XF24 analyzer. D, energy metabolism (ATP/AMP, ATP/ADP, NADH/NAD) in MDA‐MB‐231 using HPLC. E, angiogenesis of MDA‐MB‐231 cells (×100). F, the tube length and the number of nodes imaged using Image J software. ^*^ *P* < .05 vs the mimic NC or probe NC group (cells transfected with mimic NC or incubated with probe NC). he measurement data were expressed as mean ± standard deviation. One‐way ANOVA was used for comparison among multiple groups. The experiment was repeated three times

To further investigate functional significance of the interaction between miR‐328‐3p and hsa_circRNA_002178, MDA‐MB‐231 cells were transfected with mimic NC alone or cotransfected with oe‐hsa_circRNA_002178 and mimic NC or miR‐328‐3p mimic. Both CCK‐8 and Western blot assays showed that the proliferation of breast cancer cells cotransfected with oe‐hsa_circRNA_002178 and mimic NC was higher than that in cells cotransfected with oe‐hsa_circRNA_002178 and miR‐328‐3p mimic (*P* < .05), suggesting that hsa_circRNA_002178‐induced elevation in cell proliferation was reversed by increasing miR‐328‐3p (Figure 5A,B). Seahorse XF24 analyzer and HPLC found that the levels of energy metabolism markers ECAR, OCR, ATP and their metabolites increased by hsa_circRNA_002178 were eliminated by enhancement of miR‐328‐3p (*P* < .05) (Figure 5C,D). In addition, the tube formation experiments demonstrated that the number of nodes and tube length were increased by overexpression of hsa_circRNA_002178, which were neutralized by miR‐328‐3p mimic transfection (*P* < .05) (Figure 5E,F). Taken together, the data supported the conclusion that miR‐328‐3p can eliminate the promotive effect of hsa_circRNA_002178 on energy metabolism and angiogenesis.

### 3.6 Hsa_circRNA_002178 up‐regulates COL1A1 expression through sponging miR‐328‐3p

**Figure 6:**
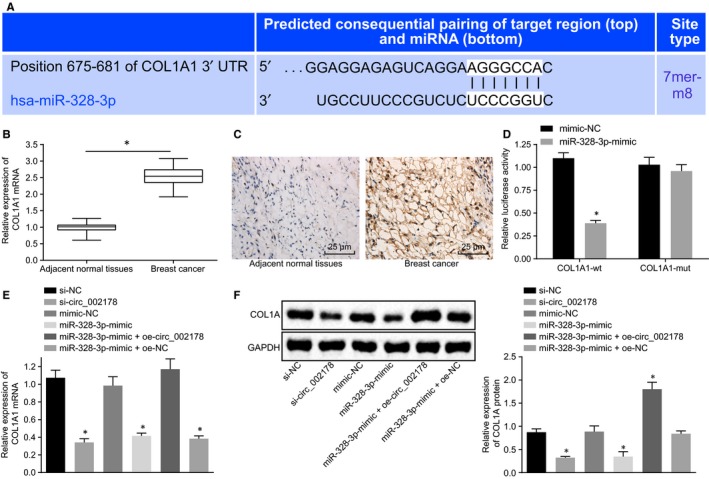
Hsa_circRNA_002178 increases COL1A1 expression through competitively binding to miR‐328‐3p. A, bioinformatics analysis showing miR‐328‐3p binding sites in 3′‐UTR of COL1A1. B, RT‐qPCR determination of COL1A1 mRNA expression in breast cancer and adjacent normal tissues, n = 70; C, protein expression of COL1A1 detected by immunohistochemical staining (× 400). D, dual luciferase activity detection for the relationship between miR‐328‐3p and COL1A1; E COL1A1 mRNA expression in MDA‐MB‐231 cells transfected with si‐hsa_circRNA_002178, miR‐328‐3p mimic, or cotransfected with miR‐328‐3p mimic and oe‐hsa_circRNA_002178 or oe‐NC. F, the protein expression of COL1A1 in MDA‐MB‐231 cells transfected with si‐hsa_circRNA_002178, miR‐328‐3p mimic, or cotransfected with miR‐328‐3p mimic and hsa_circRNA_002178‐wt or hsa_circRNA_002178‐mut measured by Western blot assay. ^*^ *P* < .05 vs the mimic NC or si‐NC group (MDA‐MB‐231 cells transfected with mimic NC or si‐NC). The measurement data were expressed as mean ± standard deviation. Unpaired *t* test was conducted for comparison between the two groups. One‐way ANOVA was used for comparison among multiple groups. The experiment was repeated three times

To further explore the mechanism by which miR‐328‐3p neutralized the cancer‐promoting effect of hsa_circRNA_002178, a series of assays were conducted to identify the relationship between hsa_circRNA_002178 and miR‐328‐3p the interaction between miR‐328‐3p and its target gene of miR‐328‐3p, COL1A1. Bioinformatics analysis suggested a miR‐328‐3p binding site in COL1A1 3′‐UTR (Figure 6A). RT‐qPCR and immunohistochemical staining characterized higher expression of COL1A1 mRNA and protein in breast cancer tissues than in adjacent normal tissues (Figure 6B,C). As shown by the dual luciferase reporter assay, the luciferase activity of COL1A1‐wt 3′‐UTR was remarkably reduced after co‐transfection with miR‐328‐3p mimic (*P* < .05), while that of COL1A1‐mut 3′‐UTR was not affected (Figure 6D), suggesting that miR‐328‐3p can specifically bind to COL1A1. To further investigate whether hsa_circRNA_002178 regulates the expression of the target gene COL1A1 by binding to miR‐328‐3p, MDA‐MB‐231 cells were transfected with si‐hsa_circRNA_002178, miR‐328‐3p mimic alone and cotransfected with miR‐328‐3p mimic and oe‐hsa_circRNA_002178 or oe‐NC. RT‐qPCR and Western blot data showed that silencing hsa_circRNA_002178 or overexpressing miR‐328‐3p inhibited the mRNA and protein expression of COL1A1 (*P* < .05), while miR‐328‐3p‐induced decrease in mRNA and protein expression of COL1A1 was rescued by oe‐hsa_circRNA_002178 (Figure 6E,F). Together, these data revealed that COL1A1 was a direct target of miR‐328‐3p, and hsa_circRNA_002178 positively regulated the expression of COL1A1 by competitively binding to miR‐328‐3p.

### 3.7 Hsa_circRNA_002178 silencing hinders tumour growth in vivo through up‐regulating miR‐328‐3p

**Figure 7:**
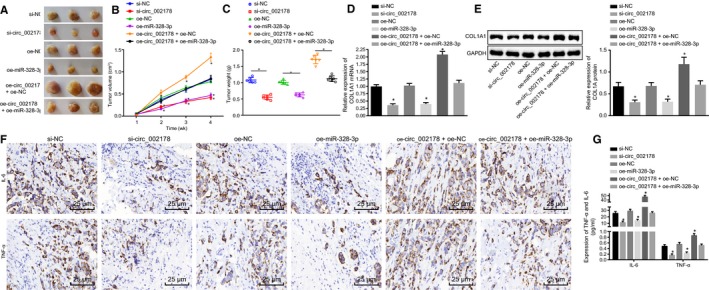
Hsa_circRNA_002178 silencing prevents tumorigenesis through increasing miR‐328‐3p expression. Stably transfected MDA‐MB‐231 cells were injected into nude mice. A, Representative images of xenograft tumours in nude mice. B, volume of tumours in nude mice. C, weight of tumours in nude mice at 4th week. D, RT‐qPCR determination of COL1A1 mRNA expression in tumour tissues of nude mice. E, COL1A1 protein expression in nude mice tumour tissue measured by Western blot assay. F, TNF‐α and IL‐6 expression in tumour tissues of nude mice detected by immunohistochemical staining (×400). G, TNF‐α and IL‐6 contents in serum of nude mice measured by ELISA. ^*^ *P* < .05 vs the si‐NC or oe‐NC group (MDA‐MB‐231 cells transfected with si‐NC or oe‐NC). The measurement data were expressed as mean ± standard deviation. One‐way ANOVA was used for comparison of data among multiple groups, and repeated measurement ANOVA for comparison of data at different time points. The experiment was repeated three times

To investigate how hsa_circRNA_002178 and miR‐328‐3p affect tumour growth in vivo*,* MDA‐MB‐231 cells stably transfected with si‐hsa_circRNA_002178, oe‐miR‐328‐3p (lentivirus vector of miR‐328‐3p overexpression), oe‐hsa_circRNA_002178, or cotransfected with both oe‐hsa_circRNA_002178 and oe‐miR‐328‐3p, respectively, were subcutaneously injected into nude mice. Tumour volume was measured weekly after injection. The tumour volume and weight were decreased by silencing of hsa_circRNA_002178 or overexpression of miR‐328‐3p, while increased by overexpression of hsa_circRNA_002178 (*P* < .05). On the other hand, overexpression of miR‐328‐3p reversed the increases in tumour volume and weight induced by overexpression of hsa_circRNA_002178 (Figure 7A‐C). RT‐qPCR and Western blot assay showed that the mRNA and protein expression of COL1A1 was decreased in response to silencing of hsa_circRNA_002178 or overexpression of miR‐328‐3p (*P* < .05). On the contrary, overexpression of hsa_circRNA_002178 increased mRNA and protein expression of COL1A1, which was abolished by enhancement of miR‐328‐3p (Figure 7D,E). At the same time, immunohistochemical staining and ELISA provided data to show that the levels of TNF‐α and IL‐6 in tumour tissues and serum were decreased in response to silencing of hsa_circRNA_002178 or overexpression of miR‐328‐3p, but increased in response to overexpression of hsa_circRNA_002178 (*P* < .05). Additionally, miR‐328‐3p neutralized the elevated levels of TNF‐α and IL‐6 induced by hsa_circRNA_002178 in tumour tissues and serum (Figure 7F,G). The above results allowed us to conclude that knockdown of hsa_circRNA_002178 inhibited tumorigenesis and inflammation in nude mice through elevating expression of miR‐328‐3p.

## 4 DISCUSSION

Targeted therapy for breast cancer remains big challenges due to high costs and risk of overtreatment.^16^ Recent studies have highlighted promising potential of circRNAs as important modulators in human cancers.^17^, ^18^ Our study conducted hsa_circRNA_002178 loss‐of‐function experiments and finally identified that hsa_circRNA_002178 functioned as a miR‐328‐3p sponge and impaired miR‐328‐3p‐targeted repression of COL1A1.

The first observation was that high hsa_circRNA_002178 expression in breast cancer was associated with poor prognosis. Knockdown of hsa_circRNA_002178 suppressed aggressive malignant behaviours including cell proliferation and angiogenesis. Similar with our findings, a recent study linked the high expression of hsa_circRNA_0006528 to advanced tumour‐node‐metastasis stage and poor outcomes in breast cancer, while inhibition of this circRNA delayed cancer development.^19^ Analogously, up‐regulation of hsa_circRNA_0072995 is determined in breast cancer cells; in contrast, the silencing of hsa_circRNA_0072995 restrained aggressive malignant behaviours in breast cancer.^20^ Moreover, hsa_circRNA_002178 silencing inhibited inflammation in vivo through reducing TNF‐α and IL‐6 levels and prevented tumour growth. Targeting angiogenesis or inflammation before clinical manifestation is in favour of preventing tumour growth and progression.^21^ Changes in metabolism driven by oncogenes are capable of controlling tumour growth through regulation of oxygen consumption.^22^ These results are consistent with our finding that hsa_circRNA_002178 silencing disrupted energy metabolism with reduced OCR and ECAR levels, accompanied with decreased ATP/AMP, ATP/ADP and NADH/NAD ratios. As a result, hsa_circRNA_002178 knockdown was able to prevent progression of breast cancer. This was further verified by our in vivo experiments showing that silenced hsa_circRNA_002178 prevents tumorigenesis.

Secondly, this study further characterized that overexpression of miR‐328‐3p eliminated the promotive effect of hsa_circRNA_002178 on energy metabolism and angiogenesis. Previously, there were only limited studies on miR‐328‐3p reporting that miR‐328‐3p could enhance the radiosensitivity of non‐small cell lung cancer cells^23^ and osteosarcoma.^24^ Moreover, miR‐328‐3p exerts as an anti‐tumour miR with potential impeding osteosarcoma cell migration through regulation of MMP16.^25^ These studies have strongly suggested that miR‐328‐3p may play important role in cancer development. This study provided evidence that miR‐328‐3p was lowly expressed in clinical breast cancer tissues. Similarly, decreased miR‐328‐3p expression was determined in endometrioid endometrial carcinoma.^26^ Consistently, transfection with miR‐328‐3p mimic in breast cancer cells leads to lowered cell motility.^13^ miR‐328‐3p was also involved in the regulation of liver protein metabolism mediated by protein homeostasis and metabolic efficiency.^27^

It was also suggested that hsa_circRNA_002178 positively regulated the expression of COL1A1 via sponging miR‐328‐3p. Recently, it has been reported that circRNAs in mammals are able to act as sponges or competing endogenous RNA (ceRNAs) of miRs thus affecting its transcriptional control.^28^ For instance, another circRNA circHIPK3 has been identified as a miR‐29b‐3p sponge effectively regulate the expression of COL1A1, which is also a target gene of miR‐29b‐3p.^29^ In addition, circCOL3A1‐859267 also binds to miR‐29c and restored the inhibition of COL1A1 induced by miR‐29c.^30^ Those findings are partially consistent with our findings that hsa_circRNA_002178 rescued miR‐328‐3p‐induced decrease in mRNA and protein expression of COL1A1. On the contrary, silencing of hsa_circRNA_002178 resulted in reduction of COL1A1 by increasing miR‐328‐3p. COL1A1 is identified as a hub gene for non‐inflammatory breast cancer.^31^ Elevated expression of COL1A1 not only correlates with advanced breast cancer and poor prognosis but also related to cancer cell invasion and metastasis.^32^ Like the finding in this study, COL1A1 acts as a target gene of miR‐196b‐5p and underlies the inhibitory effects on breast cancer cell survival and metastasis.^33^ Based on the aforementioned findings, we conclude that hsa_circRNA_002178 played inductive role in aggressive malignant behaviours of breast cancer cells through impairing the repression of COL1A1 mediated by miR‐328‐3p.

**Figure 8:**
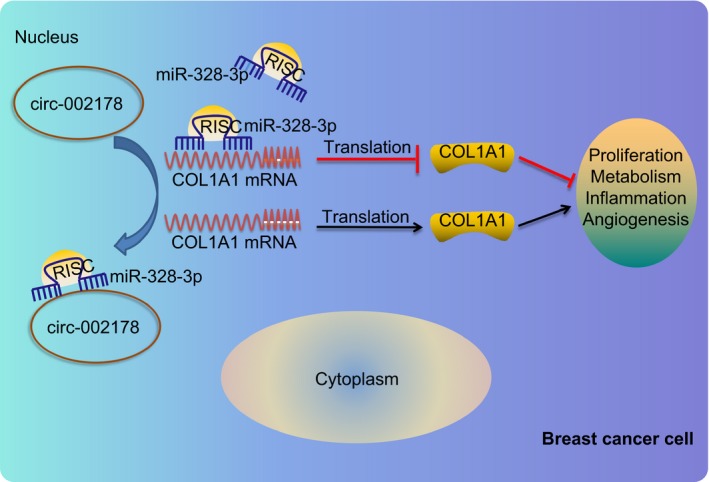
The mechanism underlining the role of hsa_circRNA_002178 in the progression of breast cancer with the involvement of miR‐328‐3p and COL1A1. In breast cancer cells, hsa_circRNA_002178 sponges miR‐328‐3p that inhibits the expression of COL1A1. The potential miR‐328‐3p targeting COL1A1 is decreased by hsa_circRNA_002178, the level of COL1A1 is increased accordingly, thereby promoting breast cancer. Inhibition of hsa_circRNA_002178 expression decreased its ability to sponge miR‐328‐3p and decreased the level of COL1A1, thereby inhibiting breast cancer cell proliferation, energy metabolism, angiogenesis and tumour inflammation. RISC, RNA‐induced silencing complex

Taken together, the findings in our paper suggests a potential therapeutic target hsa_circRNA_002178 for breast cancer treatment. A molecular network that hsa_circRNA_002178 positively regulates COL1A1 by sponging miR‐328‐3p is proposed (Figure 8), which might be conducive to the understanding of the pathological process and genetic mechanisms. Despite the model illustrated in this study, the specific metabolic pathways and pro‐ or anti‐inflammatory signals warrant future studies.

## CONFLICT OF INTEREST

None.

## AUTHOR CONTRIBUTIONS

TL and PY designed the study. TL, YYY and SL collated the data, carried out data analyses and produced the initial draft of the manuscript. PY and BSH contributed to drafting the manuscript. All authors have read and approved the final submitted manuscript.

## Data Availability

The datasets used and/or analysed during the current study are available from the corresponding author on reasonable request.
