# Supplementary material for: Circular RNA hsa_circRNA_002178 silencing retards breast cancer progression via microRNA‐328‐3p‐mediated inhibition of COL1A1
Source: J Cell Mol Med. 2020 Jan 19;24(3):2189–201. doi: 10.1111/jcmm.14875 (PMC7011152; doi:10.1111/jcmm.14875)
Supplement: Supplementary file 1 [file JCMM-24-2189-s001.docx]

**Supplementary table 1** Sequences of FISH probes.

| FISH probe | Sequence |
| --- | --- |
| hsa_circRNA_002178 | 5'- AGGGAACCCTTCCAGACTCTGATCCCGGT-3' |
| NC | 5'- GTCACTCCACTCCCATGT CCCTTG GG-3' |
