# Supplementary material for: Circular RNA hsa_circRNA_002178 silencing retards breast cancer progression via microRNA‐328‐3p‐mediated inhibition of COL1A1
Source: J Cell Mol Med. 2020 Jan 19;24(3):2189–201. doi: 10.1111/jcmm.14875 (PMC7011152; doi:10.1111/jcmm.14875)

**pGL3-Basic**

Replicon: pUC ori,f1 ori

Terminator: SV40 poly(A) signal

Vector classification: mammary cells; signaling pathway reporter vector

Vector size: 4818bp

Pronucleus resistance: Amp

Clone strain: DH5α

Culture condition: 37℃, aerobic LB

Expression host: mammary cells

Induction: Transient expression

5'sequence primer: RVP3:CTAGCAAAATAGGCTGTCCC

3'sequence primer: primers designed based on sequence


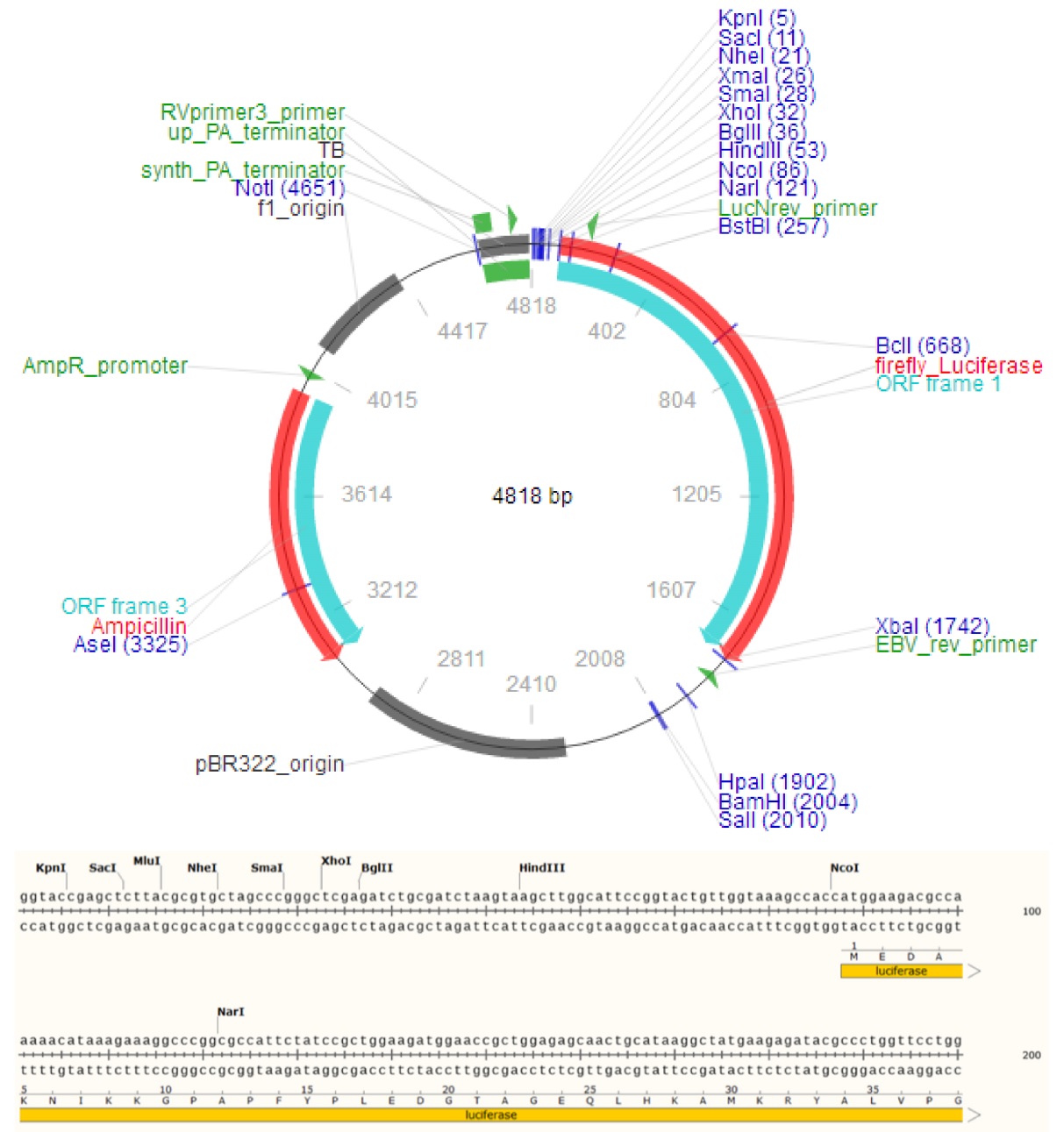

Supplement: Supplementary file 2 [file JCMM-24-2189-s002.docx]
