# Supplementary material for: Circular RNA hsa_circRNA_002178 silencing retards breast cancer progression via microRNA‐328‐3p‐mediated inhibition of COL1A1
Source: J Cell Mol Med. 2020 Jan 19;24(3):2189–201. doi: 10.1111/jcmm.14875 (PMC7011152; doi:10.1111/jcmm.14875)
Supplement: Supplementary file 3 [file JCMM-24-2189-s003.docx]

**Supplementary material 1**

**The script of R language**

#Difference analysis script

#This script is an example of using GSE101123

# Loading R Pack

source("http://bioconductor.org/biocLite.R")

biocLite("limma")

biocLite("impute")

library(limma)

library("impute")

library(data.table)

# Setting threshold

logFoldChange=1

adjustP=0.05

# Import data

setwd("C:\\Users\\lenovo\\Desktop\\GSE101123")

rt=fread("input.txt",sep="\t",header=T)

rt=as.matrix(rt)

rownames(rt)=rt[,1]

exp=rt[,2:ncol(rt)]

dimnames=list(rownames(exp),colnames(exp))

exp=matrix(as.numeric(as.matrix(exp)),nrow=nrow(exp),dimnames=dimnames)

# Processing missing data

mat=impute.knn(exp)

rt=mat$data

rt=avereps(rt)

# Draw the overall expression box chart

pdf(file="rawBox.pdf")

boxplot(rt,col = "blue",xaxt = "n",outline = F)

dev.off()

rt=normalizeBetweenArrays(as.matrix(rt))

pdf(file="normalBox.pdf")

boxplot(rt,col = "red",xaxt = "n",outline = F)

dev.off()

# Analysis of Expressional Differences

class <- c(rep("nor",3),rep("tumor",8))

design <- model.matrix(~0+factor(class))

colnames(design) <- c("nor","tumor")

fit <- lmFit(rt,design)

cont.matrix<-makeContrasts(tumor-nor,levels=design)

fit2 <- contrasts.fit(fit, cont.matrix)

fit2 <- eBayes(fit2)

# Derived differentially expressed information tables

allDiff=topTable(fit2,adjust='fdr',number=200000)

write.table(allDiff,file="limmaTab.xls",sep="\t",quote=F,col.names = NA)

diffSig <- allDiff[with(allDiff, (abs(logFC)>logFoldChange & P.Value < adjustP )), ]

write.table(diffSig,file="diff.xls",sep="\t",quote=F,col.names = NA)

diffUp <- allDiff[with(allDiff, (logFC>logFoldChange & P.Value < adjustP )), ]

write.table(diffUp,file="up.xls",sep="\t",quote=F,col.names = NA)

diffDown <- allDiff[with(allDiff, (logFC<(-logFoldChange) & P.Value < adjustP )), ]

write.table(diffDown,file="down.xls",sep="\t",quote=F,col.names = NA)

hmExp=rt[rownames(diffSig),]

diffExp=rbind(id=colnames(hmExp),hmExp)

write.table(diffExp,file="diffExp.txt",sep="\t",quote=F,col.names=F)

**The scripts of gene expression heat map of R language**

#Gene Thermal Mapping Script in R

#This script is an example of using GSE101123

# Loading R Pack

source("http://bioconductor.org/biocLite.R")

install.packages("pheatmap")

library(pheatmap)

# Import data

setwd("C:\\Users\\lenovo\\Desktop\\GSE101123")

rt=read.table("diffExp.txt",sep="\t",header=T,row.names=1,check.names=F)

rt=rt[1:nrow(rt),]

rt=rt[1:50,]

annotation=read.table("group.txt",sep="\t",header=T,row.names=1)

# Mapping gene expression thermograms

tiff(file="heatmap1.tiff",width = 15,height = 35,units ="cm",compression="lzw",bg="white",res=500)

pheatmap(rt, annotation=annotation,fontsize_row=7,fontsize_col=10)

dev.off()
